# RNA polymerase II-binding aptamers in human ACRO1 satellites disrupt transcription *in cis*

**DOI:** 10.1080/21541264.2020.1790990

**Published:** 2020-07-14

**Authors:** Jennifer L. Boots, Frederike von Pelchrzim, Adam Weiss, Bob Zimmermann, Theres Friesacher, Maximilian Radtke, Marek Żywicki, Doris Chen, Katarzyna Matylla-Kulińska, Bojan Zagrovic, Renée Schroeder

**Affiliations:** a Department of Biochemistry and Molecular Cell Biology, Max Perutz Labs, University of Vienna, Vienna, Austria; b Department of Structural and Computational Biology, Max Perutz Labs, University of Vienna, Vienna, Austria; c Department of Computational Biology, Institute of Molecular Biology and Biotechnology, Adam Mickiewicz University in Poznan, Poznan, Poland

**Keywords:** Transcription, RNA polymerase II, RNA aptamers, regulatory RNAs, silencing, repeats

## Abstract

Transcription elongation is a highly regulated process affected by many proteins, RNAs and the underlying DNA. Here we show that the nascent RNA can interfere with transcription in human cells, extending our previous findings from bacteria and yeast. We identified a variety of Pol II-binding aptamers (RAPs), prominent in repeat elements such as ACRO1 satellites, LINE1 retrotransposons and CA simple repeats, and also in several protein-coding genes. ACRO1 repeat, when translated *in silico*, exhibits ~50% identity with the Pol II CTD sequence. Taken together with a recent proposal that proteins in general tend to interact with RNAs similar to their cognate mRNAs, this suggests a mechanism for RAP binding. Using a reporter construct, we show that ACRO1 potently inhibits Pol II elongation *in cis*. We propose a novel mode of transcriptional regulation in humans, in which the nascent RNA binds Pol II to silence its own expression.

## Introduction

Control of gene expression is essential for all living organisms to coordinate growth and development. Transcription, as the first step, is tightly regulated, and RNA polymerase II (Pol II) progression along the gene is not smooth. Pol II pauses in the promoter-proximal region and also during elongation [1–3]. The dynamics of the elongating Pol II vary on a gene-by-gene basis suggesting that the underlying gene sequence is a relevant factor for transcription efficiency [1,4]. A large number of protein factors regulate transcription in various ways and, recently, several RNAs have been identified that interfere with transcription via diverse mechanisms, either indirectly (*e.g*., long non-coding RNAs affecting transcription by changing chromatin structure and function [5]), or by directly interacting with the transcription machinery [6–8]. To date, only two naturally occurring *trans*-acting RNAs have been reported to directly bind to RNA polymerase and inhibit transcription in eukaryotes [7,8]: mouse B2 and human Alu RNAs are induced by stress [9] and downregulate initiation of Pol II transcription at promoters [8]. In addition, an *in vitro* selected RNA, the FC aptamer, is able to inhibit transcription of yeast Pol II *in vitro* by binding to the active center cleft [10]. Certain RNAs are also able to serve as a template for an ancient RNA-dependent RNA polymerase activity of Pol II [11–13].

A less-explored field is the impact of *cis*-acting nascent RNA-borne signals on transcription. Bacterial riboswitches, located in the 5ʹ untranslated regions of mRNAs, can dynamically refold in response to ligand binding or temperature shift and promote transcription elongation or termination [14,15]. Similarly, eukaryotic Pol II activity has been shown to be affected by secondary structure in the nascent RNA. Specifically, stable structural elements inhibit backtracking, which leads to decreased rate of pausing and increased rate of transcription [16]. Furthermore, nascent RNAs can bind and trap transcription factors to the site of transcription contributing to their association with cognate DNA elements [17]. Alternatively, nascent transcripts can recruit proteins that cause transcription attenuation [15]. For example, the recognition motifs of Nrd1 and Nab3, components of a yeast transcription terminator complex are enriched in ncRNAs but depleted from mRNAs [15,18].

Our laboratory has shown that nascent RNA can regulate transcription by direct binding to the transcribing polymerase in *Escherichia coli* and *Saccharomyces cerevisiae* [19,20]. Short CA-rich elements within the emerging RNA, which we called RNA polymerase-binding aptamers (RAPs), potently attenuate transcription of their host genes, or increase the expression of antisense genes by suppressing transcriptional interference [21].

In this work, we extend the findings from bacteria and yeast and show that human Pol II is amenable to regulation by RAPs in the nascent RNA as well. We performed a genomic SELEX experiment to look for RAPs encoded in the human genome. We focus on one of the most highly enriched SELEX sequences derived from ACRO1 satellites and show that ACRO1-derived RAPs are potent self-silencing elements.

## Results

### Genomic SELEX identifies Pol II-binding aptamers encoded in the human genome

**Figure 1.**
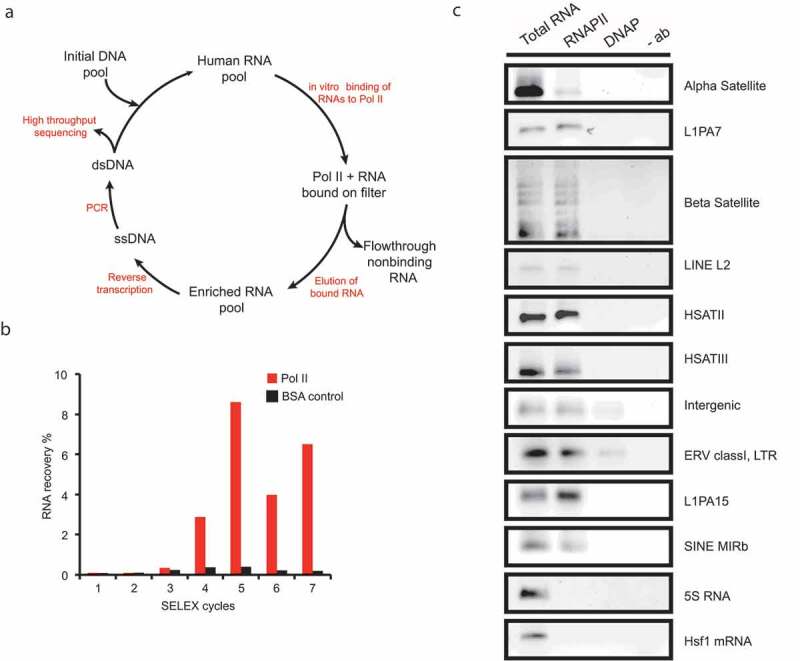
Genomic SELEX for RNA polymerase II-binding elements (RAPs)

We constructed an RNA library [22] representing the human genome in short (30–400 nt) transcripts and screened it for high-affinity binding to a purified complete Pol II 12-subunit complex from *S. cerevisiae*, since human Pol II could not be obtained in sufficient purity and quantity. Due to the high degree of conservation of the enzyme [23] and the fact that murine B2 RNA is able to bind to the *S. cerevisiae* Pol II core [24], we assumed that the binding sites for other RNAs might also be conserved. In the course of the SELEX procedure (Figure 1(a)), Pol II-binding RNAs started to enrich in the 4th cycle (Figure 1(b)) showing that the vast majority of RNAs in the starting pool do not bind to Pol II. We enforced higher stringency in the 6th and 7th cycles by lowering the protein concentration, thereby increasing the RNA-to-protein ratio in order to select sequences that bind in the low nanomolar range.

(a) The initial human DNA library was *in vitro* transcribed and the resulting RNA pool was bound to the highly purified yeast Pol II. Protein-bound RNAs were retained on the filter and non-binding RNAs were discarded. Selected RNAs were eluted from the filter and reverse transcribed into DNA. After PCR amplification, the resulting cDNA pool was subjected to another cycle of SELEX. After sufficient enrichment, the pool can be either cloned and individually sequenced or subjected to parallel sequencing [22]. (b) Enrichment of Pol II-bound human RNAs is shown for each SELEX cycle. The percentage of the recovered RNA was calculated in relation to the input RNA (red bars). In cycles 1–5 a 10:1 molar excess of RNA over protein was used, whereas in cycle 6 and 7, the RNA-to-protein ratio was increased to 100:1. BSA was used as a negative control (black bars). (c) To validate binding of selected RNAs to human Pol II *in vivo*, lysate of heat-shocked HeLa cells was co-immunoprecipitated with RNA Pol II- or DNA polymerase-specific antibodies and subjected to RT-PCR. 5S and Hsf1 are abundant cellular RNAs used as control that were not enriched by SELEX.

We selected 200 clones from the 7th cycle for Sanger-sequencing which resulted in 74 individual RNAs. We validated the selection by showing that a set of exemplary RNAs from the 7th SELEX cycle are expressed in HeLa cells (SI Fig. S1A), bind human Pol II *in vitro* (SI Fig. S1B) and can be co-immunoprecipitated with Pol II from HeLa lysates (Fig. 1(c)), vindicating our assumption that yeast and human Pol II share cognate RNAs. The predominant RNA species among the 200 individually cloned aptamers were derived from repeat regions, such as LINEs, SINEs and satellites. These findings show that the successfully selected RNAs bind to Pol II in their natural context. Binding of total RNA from the 7th cycle pool to purified human Pol II can be partially outcompeted by B2 RNA; thus, a fraction of RAPs presumably interact with the Pol II active site (SI Fig. S1C) [24].

### RAPs are found throughout the human genome, most notably in repeat regions

**Figure 2.**
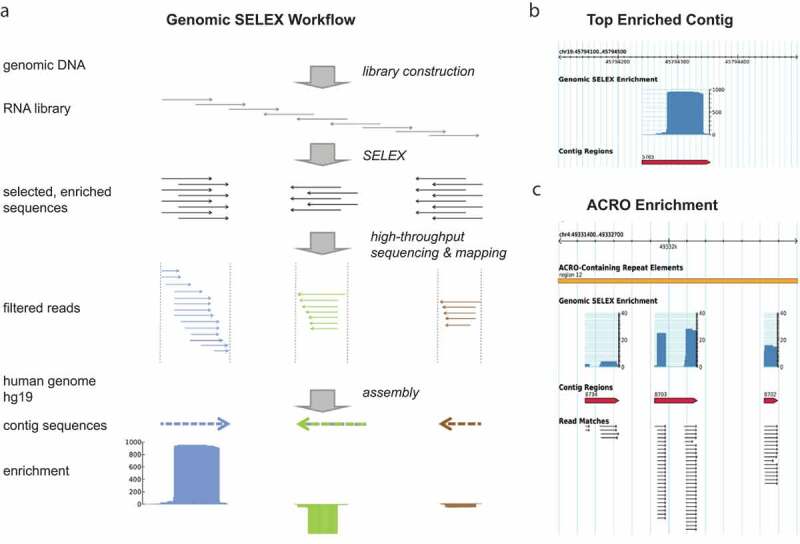
Schematic of the workflow for the selection and the analysis of RAPs

Although the selection procedure resulted in the successful isolation of RNAs binding to Pol II, no significantly enriched sequence was observed in the small sample of 200 clones, suggesting that the pool from the 7th cycle contained many diverse sequences. Therefore, we subjected this enriched pool to deep sequencing and computational analysis (Figure 2). Enriched RNAs were mapped uniquely or multiple times to the genome (SI Fig. S2A). The unique hits were enriched in genic and intergenic regions, in sense as well as antisense orientation relative to the coding strand. The most prominently enriched RAP 5765 maps to the sense strand of intron 13 of the *MARK4* gene on chromosome 19 (Table 1). The majority of sequences, however, mapped to repeat regions and their enrichment was normalized according to their frequency in the human genome (Table 2). The enriched RNAs did not contain one single dominant sequence or structural motif, suggesting that Pol II can bind a variety of diverse RNA molecules. Generally, RAPs were more CA-rich than expected by chance (SI Fig. S2B), which is consistent with our observations in *E. coli* and *S. cerevisiae* [19,20], and the highest enrichment score among the repeats was reached by (CAC^A^/_T_^C^/_A_)_n_ simple repeats and the ACRO1 family of satellites.

(a) A human RNA library was constructed and selected for RNAs binding to Pol II. The enriched pool from the 7th cycle was subjected to 454 sequencing and later the pool from the 6th cycle was Solexa sequenced. The obtained reads were filtered, mapped to the human genome (hg18 and hg19) and annotated to contigs of 400 nt in length. (b) Top enriched RAP 5765. Typical read stacks mapped and annotated to the human genome and displayed as custom track in the UCSC Genome Browser (http://genome.ucsc.edu/cgi-bin/hgGateway). (c) Enrichment of RAPs in human ACRO1 satellites was weighed and normalized to their frequency in the genome (blue bars). Arrows represent individual sequenced reads. Many of the ACRO1-associated RAPs map to the ACRO-rich centromeric region of chromosome 4. Each arrow corresponds to one read, its direction indicating the sequence orientation compared to the reference genome (plus strand)

**Table 1.** Top uniquely mapped genic PBEs

PBE ID	Gene	Chromosome	Read count	Length (nt)	Orientation^a^
5765	Microtubule affinity-regulating kinase 4 (MARK4)	19	948	113	Sense
141	Histone deacetylase 1 (HDAC1)	1	674	51	Anti
858	Microtubule affinity-regulating kinase 1 (MARK1)	1	594	42	Anti
10,384	Guanylyl cyclase-activating protein 1 (GUCA1A)	6	535	58	Anti
933	Probable saccharopine dehydrogenase (SCCPDH)	1	401	105	Sense
2312	Voltage-dependent L-type calcium channel subunit alpha-1 C (CACNA1 C)	12	261	91	Anti
122	Sodium/hydrogen exchanger 1 (SLC 9A1)	1	247	60	Anti
6885	Disintegrin and metalloproteinase domain containing protein 33 (ADAM33)	20	244	40	Sense
5920	Hippocalcin like protein 1 (HPCAL1)	2	92	34	Sense
90	Immunoglobulin superfamily member 21 (IGSF21)	1	86	58	Anti

^a^relative to mRNA strand

**Table 2.** Top repeat-derived RAPs

		Number of Mappings			
Repeat type	Repeat family	Sense	Antisense	Fold enrichment^a,b^	
Simple repeat	(CACAC)n	1393	265	2152	
Satellite	ACRO1	1029	1	1274	
Simple repeat	(CACCAT)n	2020	20	944	
LINE1	L1HAL-2a MD	2498	8	566	
DNA	hAT-Charlie	118	0	202	
	(CA)n	12,762	1800	131	
…					
LINE	L1HS^c^	214	94	5	

^a^enrichment of the more prominent strand normalized to the abundance in the genome^a^ enrichment of the more prominent strand normalized to the abundance in the genome

^b^the enrichment should be understood as approximation, since the number of repeat loci is unlikely to be the same in the source and reference genomes^b^ the enrichment should be understood as approximation, since the number of repeat loci is unlikely to be the same in the source and reference genomes

^c^L1HS is listed here because of its regulatory properties described previously (see text)^c^ L1HS is listed here because of its regulatory properties described previously (see text)

### ACRO1 satellites

**Figure 3.**
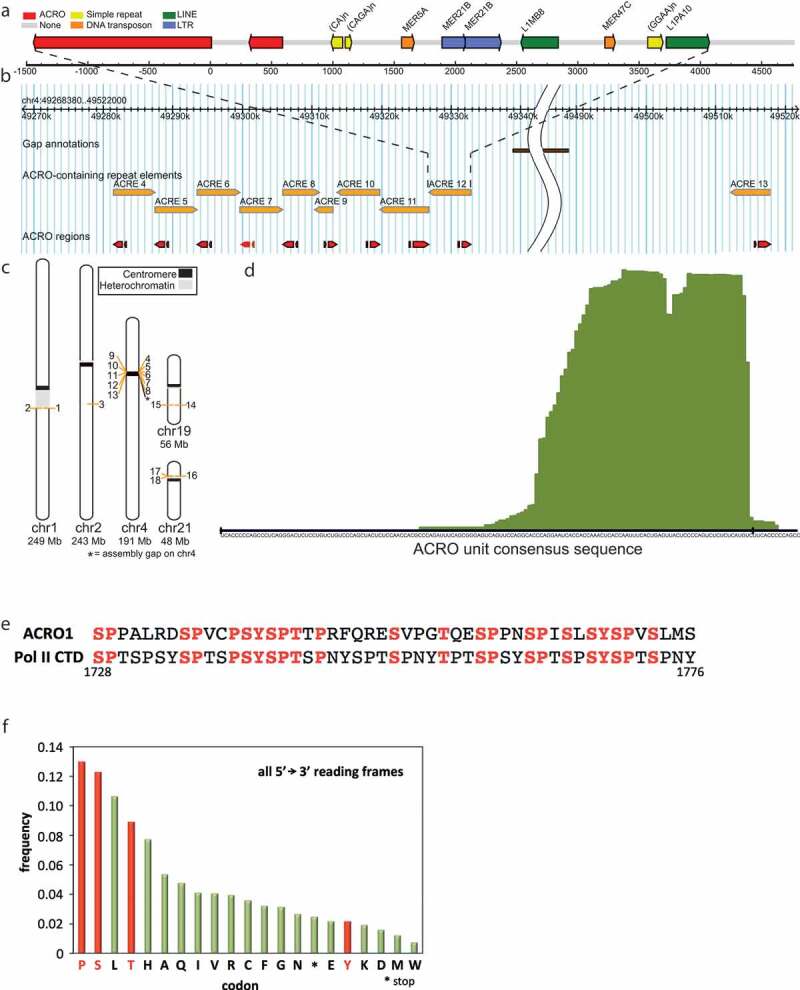
The structure and distribution of ACRO1 satellites

The ACRO1 consensus repeat unit is 147 bp long and occurs as 1.3–2.4 kb and 256 bp long arrays within a 6 kb higher-order repeat structure containing portions of LINEs, LTRs and DNA transposons. We termed these higher-order repeats “ACREs” for ACRO1-containing repeat elements (Figure 3(a) and SI Fig. S3A). While ACREs are partially or fully conserved among all sequenced primates (SI Fig. S3B), no non-primate organism was found to carry a homologue of the ACRO1 repeat. ACRO1 satellites are moderately abundant, tandem paralogue repeat elements clustered in the pericentromeric region of chromosome 4 and dispersed on chromosomes 1, 2, 19 and 21 (Figure 3(b,c)). In addition, many ACRO1 satellites have been mapped by FISH to chromosome 3 and to the acrocentric chromosomes 13, 14, 15, and 22 [25], but these regions remain to be annotated. Figure 3(d) shows SELEX read stacks mapping to the ACRO1 consensus unit defining the Pol II-binding region. These read stacks cover the ACRO1 RAPs, which are not individual *bona fide* transcripts, but rather domains within longer RNAs with Pol II-binding potential. We were unable to detect stable transcripts derived from ACRO1 satellites in HeLa cells. Nevertheless, ACRO1 satellites have been reported to be expressed at very low levels in several epithelial cancers [26] and early-stage human embryos [27].

(a) ACRE (ACRO-containing repeat element) is a higher-order repeat structure of 6 kb harboring the ACRO satellite array. (b) Organization of the ACRE cluster in the pericentromeric region of chromosome 4, the densest region of sequenced ACREs. Note that (a) shows consensus ACRE, not specifically ACRE 12. (c) ACREs were found on chromosomes 1, 2, 4, 19 and 21. (d) Sequence of ACRO1 consensus repeat unit and its SELEX enrichment profile. (e) Alignment of a translation of the consensus ACRO1 sequence with the human Pol II CTD (residue range given) with identical residues outlined in red. (f) Frequency of different codons in RAP sequences in all 5ʹ→3ʹ reading frames.

We noticed that ACRO1 satellites are rich in codons for amino acids present in the Pol II subunit 1 CTD, especially proline, serine and threonine. When the ACRO1 consensus sequence was translated *in silico* into protein and aligned globally with a fragment of Pol II CTD, 23 out of 49 amino acids were identical (e-value = 1.9 x 10^−15^, see Methods) and most convincingly also reflected the repetitive nature of the heptapeptide repeat (Figure 3(e)). Furthermore, the ACRO1 RAPs harbor part of the sequence previously identified in a random SELEX experiment that binds to the Pol II CTD with an estimated K_D_ of 600 nM [28]. This is reminiscent of the stereochemical hypothesis of genetic code origin that suggests that the code evolved in part from direct binding preferences between amino acids and their codons [29–31]. Recently, we have extended this hypothesis to suggest that proteins, especially if unstructured, might in general bind specifically to RNAs that share codon composition with their mRNAs [32–35]. We therefore also translated all the enriched human RAPs into amino acids in all three 5ʹ→3ʹ reading frames and, surprisingly, found a strong bias for amino acids proline, serine and threonine, which are present in the Pol II CTD heptapeptide repeat YSPTSPS (Figure 3(f)). The high statistical significance of this bias (p-value < 10^−23^) was ascertained by comparing the RAP-derived amino-acid frequencies to those derived from random RNA sequences, all normalized to the respective number of codons in the genetic code (see Methods for details).

### LINE1 retrotransposons are rich in RAPs

**Figure 4.**
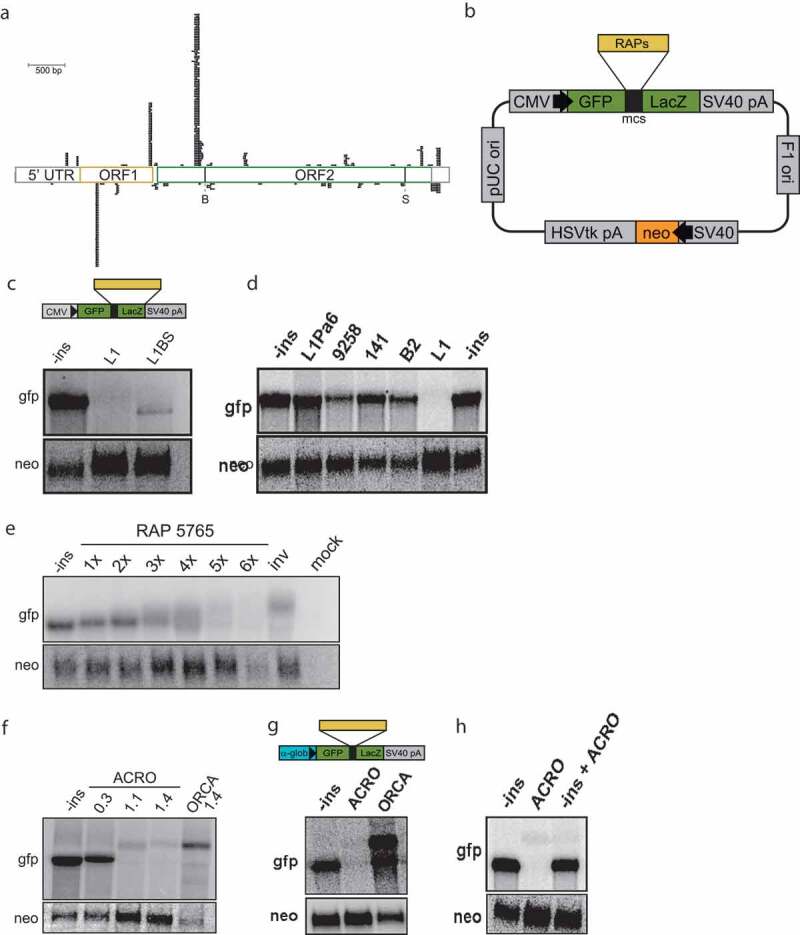
RAPs induce transcriptional silencing

Another class of repeats prominent in our selection were the LINE elements, which was especially interesting because they had previously been reported to disrupt their own expression [36]. There are multiple RAPs located within the 4 kb LINE1 ORF2 sequence (Figure 4(a)). LINEs were shown to inhibit transcription when introduced into a reporter construct (Figure 4(b)) and transfected into HeLa cells [36]. In the study by Han *et al*., it was not possible to narrow down the sequences responsible for disruption of transcription, though the effect was clearly dependent on the length of the LINE sequence. These results possibly indicate that LINEs contain sequences reducing their expression to avoid active invasion and damage of the genome caused by retrotransposition. The fact that RAPs were especially enriched in active full-length LINEs supports this hypothesis (SI Fig. S4). We repeated the above-mentioned experiments and analyzed the role of RAPs in LINE silencing. As can be seen in Figure 4(c), the presence of ORF2 abrogated transcription of the reporter gene (L1), and elimination of the flanking RAPs led to a partial recovery (L1BS). These results corroborate the notion that sequences within the LINE1 ORF2 interfere with its expression. The fact that these sequences were enriched in the SELEX experiment suggests that the silencing is mediated by interaction with Pol II.

(a) The LINE1 retrotransposon is illustrated here with the restriction sites “B” and “S” indicated [36]. LINE1-associated RAPs from the 7^th^ SELEX cycle were mapped to the consensus with at least 80% identity. (b) Vector used to monitor *in vivo* expression of the reporter cassette (adapted from [36]). RAPs or control sequences were cloned between the *GFP* and the *LacZ* sequences or, in case of L1 and L1BS, in place of LacZ gene. (c–h) Northern blot analyses of total RNA extracted from HeLa cells transfected with various RAP-containing reporters show RNA levels of the reporter gene (gfp) and a transfection control (neo). The minor bands visible especially in (e), lanes 5–8, probably derive from unspecific hybridization to 28S rRNA. (c) The cassettes contained empty GFP-LacZ fusion (-ins), LINE1 ORF2 (L1), its shortened version trimmed to the region between the “B” and “S” sites (L1BS). Note that constructs containing L1 and L1BS lack the LacZ gene so as to make the products comparable in size. (d) Diverse RAP sequences cloned into the reporter system. Apart from the single ACRO unit (9258), which had about a threefold decrease compared to no-insert plasmid (-ins), none of the other RAPs had an effect on the transcript levels. B2 RNA, which interferes with transcription *in trans*, was tested as a control. (e) RAP 5765 cloned in tandem one to six times and six times in reverse complement (inv). (f) ACRO1 as 0.3 kb, 1.1 kb and full 1.4 kb elements and its reverse complement (ORCA). (g) CMV promoter that drives expression of the reporter cassette was replaced with alpha-globin promoter. (h) To test whether presence of ACRO element affects reporter expression on a different plasmid, cassettes with empty GFP-LacZ fusion (-ins) and with full-length ACRO1 element (ACRO) were co-transfected into HeLa and expression was assessed by Northern blot.

### RAPs disrupt transcription in cis

Encouraged by this observation, we used the same system to test whether highly enriched RAPs, such as ACRO1 repeats and RAP 5765, could also lead to transcriptional disruption. Single RAPs inserted into the *GFP-LacZ* reporter system had no or only a minor effect on steady-state RNA levels (Figure 4(d)). However, insertion of multiple ACRO1 repeat units into the reporter resulted in a strong transcriptional disruption. A short insert of 0.3 kb containing two ACRO1-derived RAPs already had a visible effect, and ACRO1 insertion of 1.1 and 1.4 kb almost completely eliminated the RNA product (Figure 4(f) and SI Fig. S5). When multiple RAPs of the highly enriched genic 5765 aptamer were cloned in tandem, they severely disrupted transcription of the GFP reporter and the number of RAPs correlated with the extent of transcriptional repression (Figure 4(e) and SI Fig. S5). This down-regulating effect of the RAPs was alleviated when reverse complement sequences were used as controls confirming sequence and/or structural specificity and ruling out the possibility that a *trans*-acting DNA-binding factor constitutes a roadblock to transcription.

We further focused our analysis on ACRO1 satellites and asked whether the promoter has an impact on the transcriptional downregulation mediated by RAPs. Replacing the CMV with the alpha-globin promoter in the *GFP-LacZ* reporter resulted in similarly depleted GFP expression levels (Figure 4(g)). In addition, co-transfecting both ACRO1 and control plasmids led to full RNA levels indicating that RAPs did not have an effect on the cognate locus *in trans* (Figure 4(h)). It is thus possible that RAPs either regulate their expression co-transcriptionally or affect the stability of the mature RNA.

**Figure 5.**
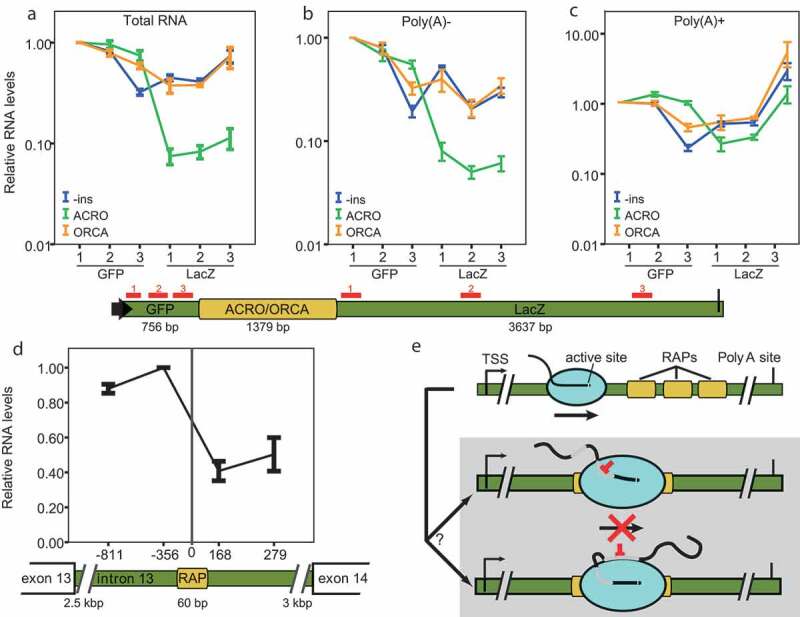
Autoregulation of RAPs is co-transcriptional

To distinguish between post- and co-transcriptional regulation, we monitored transcript levels upstream and downstream of the ACRO1 insertion by RT-qPCR (Figure 5(a)). We compared the amount of RNA three loci upstream and three loci downstream of the ACRO1 insert. The decrease of the downstream RNA levels in ACRO1-containing construct, but not in reverse complement (ORCA) or no-insert (-ins) controls, indicates that RNA production was compromised at the ACRO1 locus. Next, we repeated the experiment with separated Poly(A)+ and Poly(A)- fractions of total RNA (SI Fig. S6A). We reasoned that the Poly(A)- fraction contained incomplete products of ongoing transcription and could thus uncover true co-transcriptional regulatory events, whereas the Poly(A)+ fraction contained full-length RNAs that escaped the regulation (SI Fig. S6B). Indeed, the RNA profile in the Poly(A)+ fraction was comparable between ACRO1 construct and controls, but the downstream RNA strongly decreased in the Poly(A)- fraction indicating that RAPs have no impact on the fate of the mature full-length transcript (Figure 5(b,c)). These results show that the RAP-mediated inhibition is co-transcriptional, spatially restricted to the vicinity of the RAP template and that RAP-containing RNAs are stable once fully transcribed.

(a–c) RT-qPCR quantification of six different amplicons along the reporter transcript. Total RNA was isolated from HeLa cells 24 h after transfection with vectors carrying no insert (blue lines), ACRO (green lines) or its reverse complement ORCA (orange lines) inserts. In (b) and (c) RNA was further fractionated according to the presence (+) or absence (-) of the Poly(A) tail. All values are plotted on a log scale relative to GFP 1, the 5ʹ-most amplicon. Note different scale in (c). Error bars represent SEM of five (total RNA) and four (fractionated RNA) experiments. Transfection was controlled for by normalizing expression values to neo and subsequently, all amplicons were normalized to GFP 1. The positions of the amplicons are indicated by red bars below the panel. The reporter gene is a part of the vector from Figure 3(b). (d) RT-qPCR quantification of four amplicons surrounding the endogenous RAP 5765. Distance of the amplicon from the RAP (in bp) is indicated. Values are plotted on a linear scale relative to amplicon −356. Error bars represent SEM of three experiments. (e) Model of transcriptional inhibition by RAPs. Pol II initiates at transcription start site (TSS) and continues into productive elongation. When RAPs are present in the nascent transcript, they bind Pol II–its CTD, active site or elsewhere–rendering it elongation-incompetent. Presumably, the transcript then lacks a polyA signal and is eliminated from the cell. Note that the combined action of several RAPs might be needed for efficient regulation.

To test whether individual RAPs exert transcriptional repression in their endogenous context, we took the same approach to quantify transcript levels upstream and downstream of the most highly enriched genic RAP 5765 within the *MARK4* gene intron 13 (Figure 5(d)). The results show a moderate decrease of downstream RNA indicating that even a single RAP can modulate transcriptional output in its endogenous context.

## Discussion

### Genomic SELEX is a powerful tool to extract silencing information from genomes

Transcription is a central process in cellular life, and its regulation occurs at multiple levels. The number of proteins known to interfere with this process is large. Recently, we showed that RNA can also be a potent regulator of transcription, and that the nascent RNA contains signals that communicate with the transcription machinery in bacteria and yeast [19,20]. Genomic SELEX using the complete genomic DNA as source of RNA and purified RNA polymerase as bait proved to be a powerful approach in this context because this procedure is unbiased and also includes DNA sequences that are expressed at a very low level or not at all *in vivo* [37]. Using genomic SELEX and human genomic DNA we identified a large number of human Pol II aptamers (RAPs) encoded throughout the human genome both in unique and, most prominently, in repetitive elements. RAPs do not constitute a single RNA family with one common motif or structure, although they are generally CA-rich. RAPs are very diverse suggesting that there are many different ways that RNAs can interact with Pol II, perhaps not surprisingly, as the Pol II complex is very large and contains many potential interaction sites on its surface and in its active site. The yeast Pol II active center has been shown to be very flexible and able to accommodate quite large RNAs [24], and a cryo-EM analysis of the mammalian Pol II showed high degree of similarity between the two enzymes [38]. It should be noted, however, that by using the yeast Pol II as bait we might have missed human RAPs that do not bind to fragments conserved in the two homologues.

### When translated, ACRO1 satellites resemble Pol II CTD sequence

Recently, we have demonstrated that nucleobase-density profiles of typical mRNA coding sequences match closely the nucleobase-affinity profiles of their cognate proteins, with anti-matching seen only in the case of adenine profiles [32–35]. This finding generalized the stereochemical hypothesis of the origin of the genetic code [29–31], and suggested that proteins, especially if unstructured, may bind in a co-aligned, complementary fashion to their cognate mRNAs, but also other RNAs that share features with their mRNAs [32–35]. In direct support of this proposal, here we could show that, remarkably, ACRO1 satellites encode a protein sequence similar to the Pol II CTD and that, in addition, the RAPs are enriched in codons for the amino acids proline, serine and threonine, which feature heavily in the Pol II CTD sequence (Figure 3(e,f)). This, in turn, allows us to propose that the mechanism of RAP binding to Pol II may in part involve direct interactions between the codons contained in RAPs with their corresponding amino acids in Pol II and, especially, its CTD. Further analysis of these exciting possibilities is warranted.

Furthermore, it is possible that ACRO1 repeats are evolutionarily derived from the Pol II CTD mRNA to introduce an additional level of transcription regulation close to centromeres. ACRO1 elements are moderately abundant in the human genome and are mainly located in pericentromeric regions which are transcriptionally inactive. Their mobility could have been provided by the mobile elements contained within the ACREs (SI Fig. S3A). This would be a very recent acquisition as they can only be found in primates.

If ACRO1 RNA binds to CTD because of a mutual relationship on the codon level, why does the CTD mRNA itself not appear among RAPs? As mentioned above, adenine is the only nucleotide with anti-matching density and affinity profiles, *i.e*., adenine-rich codons tend not to bind cognate residues [33,35,39]. This suggests that the affinity between codons and their cognate amino acids could be attenuated or even reversed with increasing adenine density. Indeed, the ACRO1 density profile is an inverse of that of the CTD mRNA and matches the CTD adenine-affinity profile, providing a potential explanation for why ACRO1 could bind to Pol II CTD, while the CTD’s mRNA would not (SI Fig. S7).

### RAPs represent a novel type of regulatory RNA signals

In this work, we identified a novel level of transcription regulation in human cells by showing that RNA signals on the nascent RNA can interfere with the transcribing Pol II *in cis*, abrogating transcription. It has already elegantly been shown that the secondary structure of the nascent RNA affects the rate of Pol II transcription *in vitro* by inhibiting backtracking and thus preventing the polymerase to escape from pausing [16]. RAPs are RNA sequences that were enriched in a SELEX procedure due to their virtue of binding to Pol II. They are not *bona fide* transcripts but rather domains within potentially expressed RNAs that convey Pol II-binding capacity to their host transcripts. In the context of our experiments, RAPs are part of the nascent transcript interacting with Pol II *in cis* during transcription. We observed that their effect on transcription is additive and that the more RAPs are present on the nascent RNA the stronger the inhibitory effect (Figure 4(e,f)). Most importantly, the inhibitory effect is co-transcriptional. Once the RNA is fully transcribed, RAPs have no impact either on transcription or on the stability of the transcript (Figure 5(a–c)). Based on these observations, we hypothesize that the nascent RNA can cross-talk to Pol II via many potential interaction sites on its surface, or via the CTD, and thereby disrupt transcription (Figure 5(e)).

Recently, circular intronic long noncoding RNAs were shown to accumulate at the site of transcription, associate with the elongating RNA polymerase and act as positive regulators of transcription [40]. Here we add another layer of transcriptional regulation that involves *cis*-acting sequences within the nascent transcript that affects transcription elongation. This might be an essential self-regulatory strategy for repeat elements to stay silent, enabling their survival in the genome during evolution. In addition, we hypothesize that RAP-mediated control of transcription might play a role in gene-regulatory processes, which depend on the rate of Pol II progression, such as alternative splicing and termination [41]. Indeed, several RAPs map downstream of alternative splice sites and alternative polyadenylation sites (not shown).

### RAP-mediated transcription termination is a conserved phenomenon from bacteria to yeast to humans

In this work, we have presented evidence that Pol II can “sense” the nature of transcripts by means of direct interaction and that some RNA sequences encoded in the human genome have the potential to interfere with their own transcription *in cis*. We propose a novel mode of transcriptional control in human cells, wherein the nascent RNA binds to the transcribing Pol II making it elongation-incompetent (Figure 5(e)). Similar screens have been performed for the *E. coli* genome and the bacterial RNA polymerase and the yeast *S. cerevisiae* genome and yeast Pol II [19–21]. *E. coli* RAPs cause Rho-dependent premature transcription termination by uncoupling translation and transcription, or induction of genes on the opposite strand by attenuating transcriptional interference. Likewise, yeast RAPs induce premature transcription termination demonstrating that RAP-mediated transcription interference is a conserved phenomenon. A cross-talk between the nascent RNA and the transcription machinery could provide the primary signal that determines the fate of transcripts.

## Materials & methods

### Library construction and Genomic SELEX

The genomic library was created as described previously [37], with human genomic DNA purchased from Sigma (CAS number 9007–49-2) as template. After transcribing the genomic library into RNA, the RNA pool was bound to Pol II of *S. cerevisiae* in an *in vitro* binding reaction as described in ref [22]. For the 1^st^-5^th^ cycles, RNA was added at 1 µM and protein at 100 nM. To increase stringency and competition, RNA was added at 1 µM and protein at 10 nM for the 6^th^ and 7^th^ cycles. The binding buffer contained 10 mM HEPES pH 7.25, 40 mM NH_4_SO_4_, 10 µM ZnCl_2_, 1 mM KCl, 10 mM DTT, 5% glycerol and 10 mM MgCl_2_.

### Co-immunoprecipitation

HeLa cells grown in 10 cm dishes were harvested at 80% confluence with 1 ml lysis buffer (10 mM HEPES pH 7.0, 100 mM KCl, 5 mM MgCl_2,_ 0.5% Nonidet P-40, 1 mM DTT, 100 U/ml RNAse inhibitor (Promega), 2 mM vanadyl ribonucleoside complexes solution, 25 µl/ml protease inhibitor cocktail for mammalian tissues) per 10 cm^−1^ and removed from the dish with a cell scraper. After 10 min on ice cells were centrifuged at 4°C, 1000 × *g*. Whole cell extracts were prepared for co-IP as described [42]. RNA purified from the immunoprecipitates and input RNA were analyzed by RT-PCR with the Qiagen RT-PCR kit using primers specific for the different RNAs.

### Antibodies

Pol II and DNA polymerase antibodies were purchased from Abcam (ab817/ab5408 and ab3181, respectively). Pol II antibody recognizes the phosphorylated as well as the unphosphorylated form of Pol II the enzyme. The concentration of antibodies used for immunoprecipitations was 2 µl/ml

### Transfection, microscopy and RNA preparation

HeLa cells were grown to 70–90% confluence and transfected with 0.4 mg of plasmid per cm^2^ of culture dish using Lipofectamine 2000 (Invitrogen) according to manufacturer’s instructions. After 24 h, fluorescence was monitored with AxioObserver Z1 microscope coupled to AxioCam MRm (Carl Zeiss MicroImaging) and RNA was extracted with TRI Reagent (Sigma).

### Northern blot

Total RNA was separated on a 0.8% agarose gel containing 6.7% formaldehyde, capillary-blotted onto a Hybond-XL membrane (GE Healthcare) and UV-crosslinked. ^32^P-labeled DNA probe was hybridized in ULTRAhyb-Oligo Buffer (Ambion) at 42°C overnight. The probe was 5ʹ-labeled with T4 PNK (NEB).

### Flow cytometry

GFP-positive cells were quantified by FACSCalibur (BD Biosciences) and data were analyzed in Cyflogic (CyFlo Ltd, Finland) and SPSS (IBM) software. From each sample, fluorescence of 10,000 cells was measured and only GFP-positive events, as determined by mock-transfected cell fluorescence, were taken into account.

### Poly(A) fractionation

150 pmol biotinylated Oligo(dT) (Promega) was bound for 10 min at room temperature to 0.6 ml MagneSphere® magnetic beads (Promega) prepared according to manufacturer’s instructions. 80 mg of total RNA was denatured at 65°C, 10 min, chilled on ice for 5 min and mixed with Oligo(dT)-beads solution. After 10 min incubation at room temperature, the beads were washed six times and Poly(A)+ RNA was eluted according to manufacturer’s instructions. Before washing of the beads, the first supernatant was taken as Poly(A)-RNA. Both fractions were ethanol-precipitated.

### RT-PCR and RT-qPCR

Two milligrams of total RNA or 200 ng of Poly(A)-fractionated RNA was denatured with 200 pmol of random nonamers (Sigma) at 70°C for 10 min. The reaction was split in two, one without reverse transcriptase as a control. RT was performed at 45°C for 90 min using OmniScript (Qiagen). 1/40 of the total reaction was used for PCR and approximately 1/30 was used per qPCR well. qPCR was performed in Mastercycler® realplex (Eppendorf) with HOT FIREPol® qPCR Mix (Medibena) and primers specified in Supplementary Table S1. Transfection was controlled for by normalizing expression values to neo and subsequently all amplicons were normalized to GFP 1.

### Sequence alignment

ACRO1 translation was aligned globally against a fragment of human Pol II CTD of equivalent length via Needleman-Wunsch algorithm as implemented in Expasy lalign [43] using BLOSUM62 scoring matrix [44], opening gap penalty of −12 and extending gap penalty of −2.

### Analysis of amino acid enrichment

The statistical significance of the enrichment of Pol II CTD amino acids in translated RAPs was evaluated by an analysis of random RNA sequences generated computationally using background frequencies of the four RNA nucleotides in the entire human genome. For the complete set of RAPs identified in this study, 10^6^ sets of random RNA sequences of equivalent lengths were generated. Each set was then translated using the universal genetic code and the Jensen–Shannon divergence (JSD) between the distribution of the obtained amino-acid frequencies and the distribution of amino-acid frequencies in human Pol II CTD was determined. The p-value was determined by comparing the distribution of JSD values in the case of random sequences against the RAP-Pol II CTD JSD. The RAP-Pol II CTD JSD was over 10 standard deviations lower than the average random-sequence JSD, yielding an estimated p-value < 10^−23^. As different amino acids are encoded by a different number of codons, the above analysis was performed by first normalizing the amino-acid frequencies in translated RAPs and random RNA sequences or Pol II CTD by the respective number of codons in the universal genetic code.

### Accession numbers

The ACRO1 sequence used in the reporter assay has been deposited in the Genbank with the number GenBank KF726396. The raw data are available for download on the Sequence Read Archive under BioProject accession PRJNA616423.

## Supplementary Material

Supplemental Material
