# Supplementary material for: RNA polymerase II-binding aptamers in human ACRO1 satellites disrupt transcription in cis
Source: Transcription. 2020 Jul 14;11(5):217–29. doi: 10.1080/21541264.2020.1790990 (PMC7714431; doi:10.1080/21541264.2020.1790990)
Supplement: Supplemental Material [file KTRN_A_1790990_SM0060.zip › Supplementary information/Revision-clean version-Supplementary information.docx]

**Supplementary Methods**

**Computational analysis**

We subjected the enriched pool from the 7^th^ SELEX cycle to 454 pyrosequencing and computational analysis. To confirm reproducibility, the 6^th^ round of an independent and parallel SELEX experiment was sent to Solexa sequencing. The analysis reported here was restricted to the 454 sequences from the 7^th^ round as the same sequences dominated the pool of the 6^th^ cycle. Reads were mapped using vmatch [1] to the human genome using 95 % identity as cutoff. Reads shorter than 20 nucleotides were excluded from the analysis and reads mapping more than six times to the human genome were considered repeat-derived (as assigned by EnsGene or RepeatMasker). Over 11,000 reads mapped less than six times to the genome and were considered to be deriving from unique RAPs. To analyze repeat sequences, a lower identity cut-off of 90 % was used. Approximately 58,000 reads, which mapped to 6 or more places in the genome, were assigned to the repeat class. Removal of primer and T7 promoter sequences from the 454 sequence reads was performed with the use of BLAST and custom-made scripts. Mapping of the sequences to the human reference genome (UCSC hg18, NCBI v36) was performed by BLAT [2] with parameters adjusted to the respective read lengths. Reads mapped to the genome within 400 bp of each other were subsequently grouped into “contigs”, which was the maximum clone length after the 7^th^ SELEX cycle. For normalization of the read count per contig, sequences which match n times to the genome where counted 1/n times. For analyzing the annotation of the contigs and RAP repeat regions, the Ensembl gene set [3] and the RepeatMasker [4] annotation of interspersed repeat elements were used, both downloaded from the UCSC genome database [5]. If a contig overlapped with a certain sequence element, e.g. a SINE, with >19 nt, it was assigned to the respective class. The contig lengths were subsequently weighted by their normalized read counts; percentages refer to the total contig length. For assignment of ORF2 to the L1 elements to which RAP-RNAs could be mapped, we chose to use the 6064 bp L1HS consensus sequence as a model, as it was the closest naturally occurring consensus to the sequence reported in [4] and contained full length ORF1 and ORF2. All L1-associated RAPs were aligned using segemehl [6] requiring only 70 % identity. This was done to account for the diversity of L1 sequences, and since all these RAPs were already associated with an L1 element, the positions of the RAPs genome-wide within L1 is indicated. If not otherwise stated, custom-made scripts (Java, VBA, Perl, Python) were used.

**ACRE analysis**

The ACRO1-Containing Repeat Elements (ACREs) were found by manual inspection of the patterns shown in the UCSC genome browser [5]. The instances of ACREs were determined using an in-house script that extracted ACREs based on the instances of the long and short tandemly repeated ACROs, followed by the pattern of repeat sequences that were found in ACREs from the RepeatMasker annotation [4]. An ACRE could be considered full length if the next repeat annotation was another ACRO, implying a head-to-tail ACRE instance. This resulted in 15 full-length ACREs ranging in length from 4631-7021 and 8 truncated ACREs. All ACROs annotated in the genome formed the same pattern of one long ACRO tandem repeat containing 7-15 head-to-tail oriented ACROs. This was followed by an unknown sequence frequently of length 332, with five exceptions ranging from lengths 325-362. This was followed by two more head-to-tail oriented ACRO repeats. This was followed by a conserved series of repeat annotations with some variation. The repeat annotation of all ACRE sequences is shown in Figure E6. All ACRE sequences found were aligned using ClustalW 2.1 [7]. A consensus for the ACRE sequence was found using Consensus Maker [8]. Gaps in the consensus were removed. Repeat elements were then identified in the consensus using RepeatMasker [4].

Primate genomes calJac3 (marmoset), gorGor3 (gorilla), nomLeu1 (gibbon), panTro3 (chimpanzee) and rheMac3 (rhesus) were downloaded from the UCSC genome browser [5]. The ACRE sequence was multiply aligned to all primate genomes using mLAGAN and visualized in Figure E7 using the VISTA web tool [8]. Additional homologues were searched for using the MegaBLAST web server with default parameters [9]. RAPs that had previously categorized as mapping to ACRO1 elements were mapped to the ACRO consensus in RepBase were mapped using segemehl [6] using a cutoff of 85% identity. The coverage map was computed by counting the number of reads mapping to position in the consensus.

***In vitro* transcription**

Primers with flanking T7 promoter were obtained from MWG Munich. PCR products were used as template in *in vitro* transcription reactions using RNAMaxx high yield transcription kit (Stratagene) according to manufacturer’s instructions. Transcripts were gel-purified, precipitated by the addition of 3 volumes of absolute ethanol, collected by centrifugation and dissolved in water.

**Bandshift assays**

Native gel shift assays were performed with body-labeled *in vitro* transcribed RNA and with purified RNA polymerase from *H. sapiens* (Jena Biosciences). For individual RNAs, bandshifts were performed with a Pol II prepared according to [10] and assays with the whole human RNA pool were done with Pol II from Jena Biosciences. Binding reactions contained 10 to 100 nM RNA polymerase, 5 % glycerol, 1 mM KCl, 10 µM ZnCl_2_, 10 mM MgCl_2,_ 10 mM DTT, 10 mM HEPES, 2 mM Tris pH 7.25, 1 U RNA guard (Amersham Pharmacia), 1 mg/ml BSA and 0.2 nM ^32^P-GTP labeled RNA in a total volume of 20 µl. Reactions were incubated for 30 minutes at RT, loaded onto a 4 % native gel and run in 0.5 X TBE with 5 % glycerol and 400 nM MgOAc. The gel was dried and analysed with a phosphorimager.

**Filter binding assay**

10 nM ^32^P-labeled RNA from SELEX cycle 7 was incubated with 5 nM of Pol II (Jena Biosciences), and increasing amounts of unlabeled B2 RNA (0-100 nM) in bandshift assay-buffer was added. Binding proceeded at room temperature for 30 minutes. Protein-RNA complexes were retained on a nitrocellulose filter (Schleicher-Schuell) and nonbinding RNAs were washed away. The amount of labeled RNAs retained on the filter was analysed using a scintillation counter.

**Cell culture, heat-shock treatment, RNA preparation and RT-PCR**

HeLa cells were grown at 37 °C in a 5 % CO_2_ atmosphere in DMEM medium containing 10 % fetal calf serum (PAA). Growth media contained 2 mM glutamine and a penicillin/streptomycin cocktail. Cells were heat-shocked at 45 °C for 40 minutes with subsequent recovery at 37 °C for 1 h. Control cells were maintained at 37 °C. For analysis of expression of RAP-containing repeats presented in Figure S1A, total RNA was prepared from a whole cell lysate with the PARIS^TM^ protein and RNA isolation system (Ambion) according to manufacturer’s instructions and DNA was removed by digestion with DNase I (Promega). First strand cDNA was synthesized from 2 μg total RNA using specific primers (MWG Munich) and amplified by PCR (Qiagen RT-PCR kit).

**Supplementary Figure Legends**

**Supplementary Figure S1**

**(A) Expression of selected RAP-containing RNAs.** RAP-containing RNAs were detected in HeLa cells either grown under normal conditions or subjected to heat shock. Total RNA was isolated and strand-specific expression was monitored by RT-PCR. Fwd (forward) indicates the strand that was found to bind to Pol II, and Rev (reverse) the corresponding complementary strand. 5S rRNA and Hsf1 mRNA were analyzed as controls; the expressed strand is indicated as Fwd. **(B) Binding of selected RAP-containing RNAs to Pol II.** Electrophoretic mobility shift assays were performed under similar binding conditions as the selection procedure, with *in* *vitro* transcribed individual RNAs or with the enriched RNA pool from SELEX cycle 7 and human Pol II. We included B2 RNA, which was shown in a previous study to bind at 2 nM [11], as a positive control and beta-actin and heat shock factor 1 (hsf1) mRNAs as negative controls. The results show that the selected RAPs bind to human RNA Pol II in the low nanomolar range and that Pol II does not have high affinity to RNAs in general. **(C) RAPs compete with B2 for binding to Pol II.** The total RNA from SELEX cycle 7 was analysed via filter binding with Pol II and this binding was competed with *in vitro* transcribed B2 RNA. B2 has been shown to compete with the Fc aptamer, which binds to the active site of yeast Pol II [12].

**Supplementary Figure S2**

**(A) Genomic categorization of reads enriched in the 7th cycle of Genomic SELEX.** Reads were categorized by their location relative to the ENSEMBL gene annotation of hg18. Intergenic reads are reads which do not fall into any category, including antisense intron. LINE elements are most often attributed to RAP sequences, and together with antisense exons and introns, they make up the majority of those annotated. **(B) Base composition of RAPs.** The majority (21,710) of repeat-derived RAPs belong to the class of simple repeats (CA)n and (CACAC)n. The mono-, di- and trinucleotide content of the RAP sequences clearly show a strong over-representation of CA-rich sequences.

**Supplementary Figure S3**

**(A) All annotated ACRE regions in the human genome and the repeat elements contained therein.** All ACRE sequences were extracted from the genome. A clear pattern emerges among them and the downstream, non-ACRO parts of the sequences are highly similar. The consensus at the bottom (also shown in Fig. 3A) was reannotated using RepeatMasker. **(B) ACRE conservation in primates.** Selected sequenced primate genomes were aligned to the ACRE sequence using the mLAGAN multiple sequence and chaining aligner and visualized using the VISTA browser. Red peaks show regions of high conservation, and the repeats within the ACRE sequence are annotated above. The ACRE sequence contains some similarity across the sequenced primates. While some do not contain the full ACRE sequence, all primates have the highly conserved shorter element containing two ACRO repeats.

**Supplementary Figure S4**

**RAPs mapping to LINE elements. (A)** Distribution of RAPs in LINE1 elements of increasing length shows that full-length LINEs are the most enriched in RAPs. **(B)** Occurrence of RAPs in different LINE families shows that active LINEs are more rich in RAPs than inactive LINEs.

**Supplementary Figure S5**

**GFP expression in RAP-containing reporter cassettes. (A)** HeLa cells were transfected with plasmids from figure 4b containing various RAPs and fluorescence was monitored after 24 h. The cassettes contained empty GFP-LacZ fusion (-ins), RAP 5765 cloned in tandem three times (3x), six times (6x) and six times in reverse complement (inv), ACRO1 1.4 kb element (ACRO), its reverse complement (ORCA) and LINE1 ORF2 (L1). Mock indicates no-plasmid transfection. Cells were monitored with both phase (upper panels) and fluorescent (lower panels) microscopy; bar = 200 μm. **(B)** Quantification of GFP expression by flow cytometry. 10,000 cells from each sample were analysed 24 h post-transfection and their fluorescence levels were determined on a 1024-channel scale (fluorescence intensity). Only GFP-positive cells (determined by comparison with mock-treated sample) are plotted (count). Total number of GFP-positive cells relative to "–ins" (-ins = 1) is indicated ± SEM of five experiments.

**Supplementary Figure S6**

**(A) Poly(A)-fractionation of total RNA.** RNA isolated from HeLa cells 24 h after transfection with vectors containing empty GFP-LacZ fusion (-ins), ACRO or its reverse complement (ORCA) was fractionated into Poly(A) tail-containing (pA+) and Poly(A) tail-devoid (pA-) pools. Efficiency of this process was monitored by RT PCR and agarose gel electrophoresis using primers for GAPDH (which is polyadenylated) and 5S rRNA (which is not polyadenylated). Reactions were carried out with (+) or without (-) reverse transcriptase. Ladder is in the last lane of each panel with sizes in bp indicated rightmost. **(B)** The levels of GFP1 RNA (see figure 4) relative to the neo transfection control is compared between constructs and RNA fractions. Numbers indicate fold difference between ACRO and ORCA mean values. The observation that the difference is largest in Poly(A)+ fraction and smallest in Poly(A)- fraction is consistent with the assumption that Poly(A)- fraction contains predominantly nascent RNA, and Poly(A)+ fraction contains mature RNA (ACRO-containing RNA should be underrepresented in Poly(A)+ pool as judged from the Northern blot experiments presented in Figure 4).

**Supplementary Figure S7**

**(A)** Superposition of the adenine (ADE)-density profile of a fragment of CTD mRNA (black) with the ADE-affinity profle of the corresponding CTD fragment (red) as calculated from knowledge-based nucleobase/amino-acid affinities derived by Polyansky and Zagrovic [13, 14]. **(B)** Superposition of the ADE-density profile of ACRO1 (black) with the ADE-affinity profile of the CTD fragment from part (A) (red). Both fragments correspond to the CTD stretch given in Fig. 3E. In both (A) and (B), the RNAs are smoothed using a 21-codon window, while the protein is smoothed using a 21-residue window, as done before [13, 14]. Note that this is the reason why the values for the first 10 and the last 10 resides/codons of the shown sequences are not included.

**Supplementary References**

1. Abouelhoda, M.I., Kurtz, S., and Ohlebusch, E. (2004). Replacing suffix trees with enhanced suffix arrays. J. Discret. Algorithms *2*, 53–86.
2. Kent, W.J. (2002). BLAT---The BLAST-Like Alignment Tool. Genome Res. *12*, 656–664.
3. Flicek, P., Ahmed, I., Amode, M.R., Barrell, D., Beal, K., Brent, S., Carvalho-Silva, D., Clapham, P., Coates, G., Fairley, S., et al. (2013). Ensembl 2013. Nucleic Acids Res. *41*, D48–55.
4. Smit, A., Hubley, R., and Green, P. RepeatMasker Open-3.0. 1996-2010 <http://www.repeatmasker.org>.
5. Dreszer, T.R., Karolchik, D., Zweig, A.S., Hinrichs, A.S., Raney, B.J., Kuhn, R.M., Meyer, L.R., Wong, M., Sloan, C.A., Rosenbloom, K.R., et al. (2012). The UCSC Genome Browser database: extensions and updates 2011. Nucleic Acids Res. *40*, D918–23.
6. Hoffmann, S., Otto, C., Kurtz, S., Sharma, C.M., Khaitovich, P., Vogel, J., Stadler, P.F., and Hackermüller, J. (2009). Fast mapping of short sequences with mismatches, insertions and deletions using index structures. PLoS Comput. Biol. *5*, e1000502.
7. Larkin, M.A., Blackshields, G., Brown, N.P., Chenna, R., McGettigan, P.A., McWilliam, H., Valentin, F., Wallace, I.M., Wilm, A., Lopez, R., et al. (2007). Clustal W and Clustal X version 2.0. Bioinformatics *23*, 2947–2948.
8. Frazer, K.A., Pachter, L., Poliakov, A., Rubin, E.M., and Dubchak, I. (2004). VISTA: computational tools for comparative genomics. Nucleic Acids Res. *32*, W273–9. HIV Sequence Compendium (2012). Consensus Maker.

9. Zhang, Z., Schwartz, S., Wagner, L., and Miller, W. A greedy algorithm for aligning DNA sequences. J. Comput. Biol. *7*, 203–214.

1. Dignam, J. D., Lebovitz, R. M. & Roeder, R. G. Accurate transcription initiation by RNA polymerase II in a soluble extract from isolated mammalian nuclei. *Nucl. Acids Res* 11, 1475-89 (1983).
2. Espinoza,C.A., Allen,T.A., Hieb,A.R., Kugel,J.F. and Goodrich,J.A. (2004) B2 RNA binds directly to RNA polymerase II to repress transcript synthesis. *Nat. Struct.* *Mol. Biol.*, 11, 822–829.
3. Kettenberger,H., Eisenführ,A., Brueckner,F., Theis,M., Famulok,M. and Cramer,P. (2006) Structure of an RNA polymerase II-RNA inhibitor complex elucidates transcription regulation by noncoding RNAs. *Nat. Struct. Mol. Biol.*, 13, 44–48.
4. Polyansky, A.A., Zagrovic, B. (2013) Evidence of direct complementary interactions between messenger RNAs and their cognate proteins. *Nucleic Acids Res*, 41(18):8434–8443.
5. Zagrovic, B., Bartonek, L., Polyansky, A.A. (2018) RNA‐protein interactions in an unstructured context. *FEBS Lett* 592(17):2901-2916.
