# Supplementary figures and images for: RNA polymerase II-binding aptamers in human ACRO1 satellites disrupt transcription in cis
Source: Transcription. 2020 Jul 14;11(5):217–29. doi: 10.1080/21541264.2020.1790990 (PMC7714431; doi:10.1080/21541264.2020.1790990)

Supplementary Figure S1

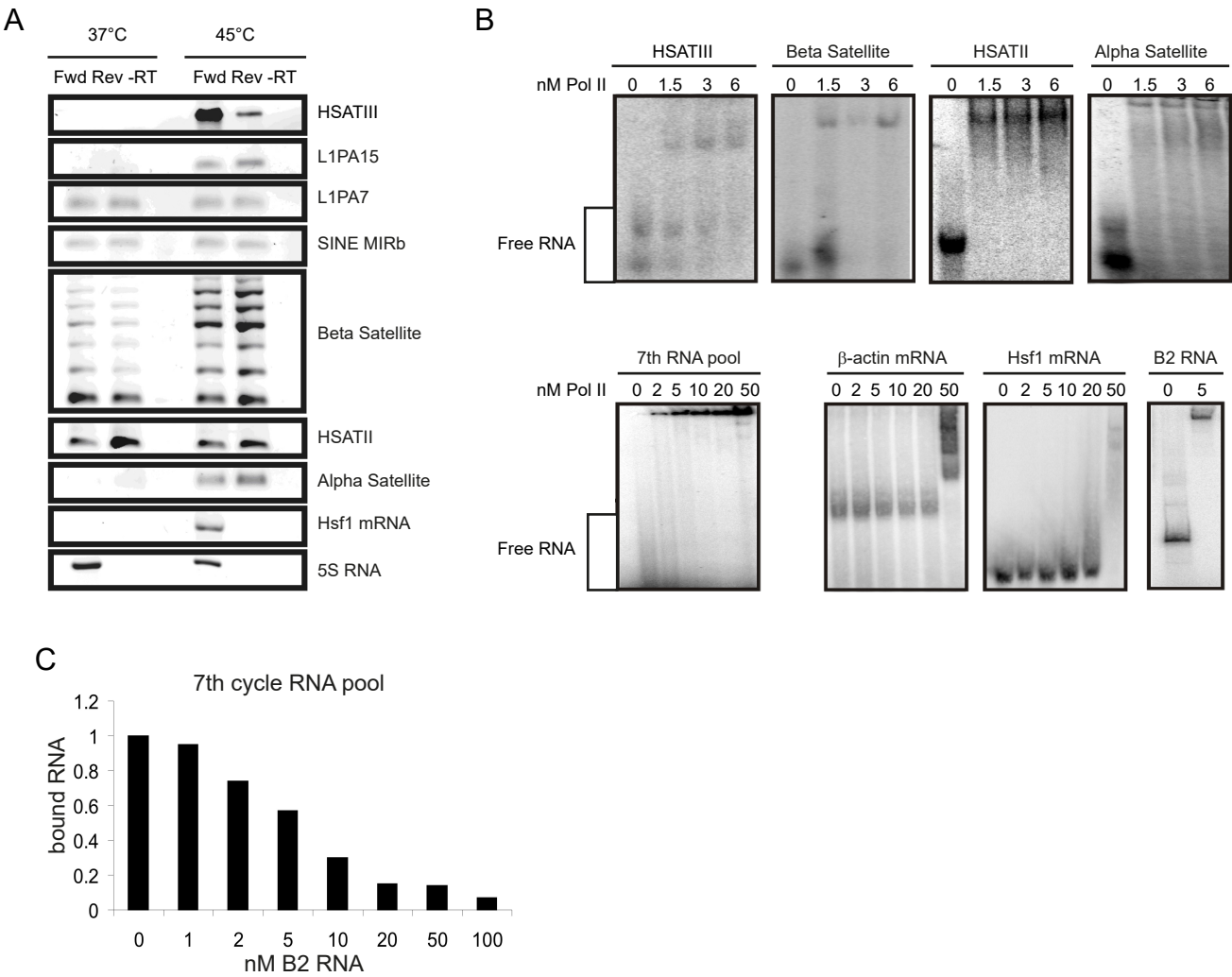

Supplement: Supplemental Material [file KTRN_A_1790990_SM0060.zip › Supplementary information/Supplementary_FigureS1.pdf]

Supplementary Figure S2

A

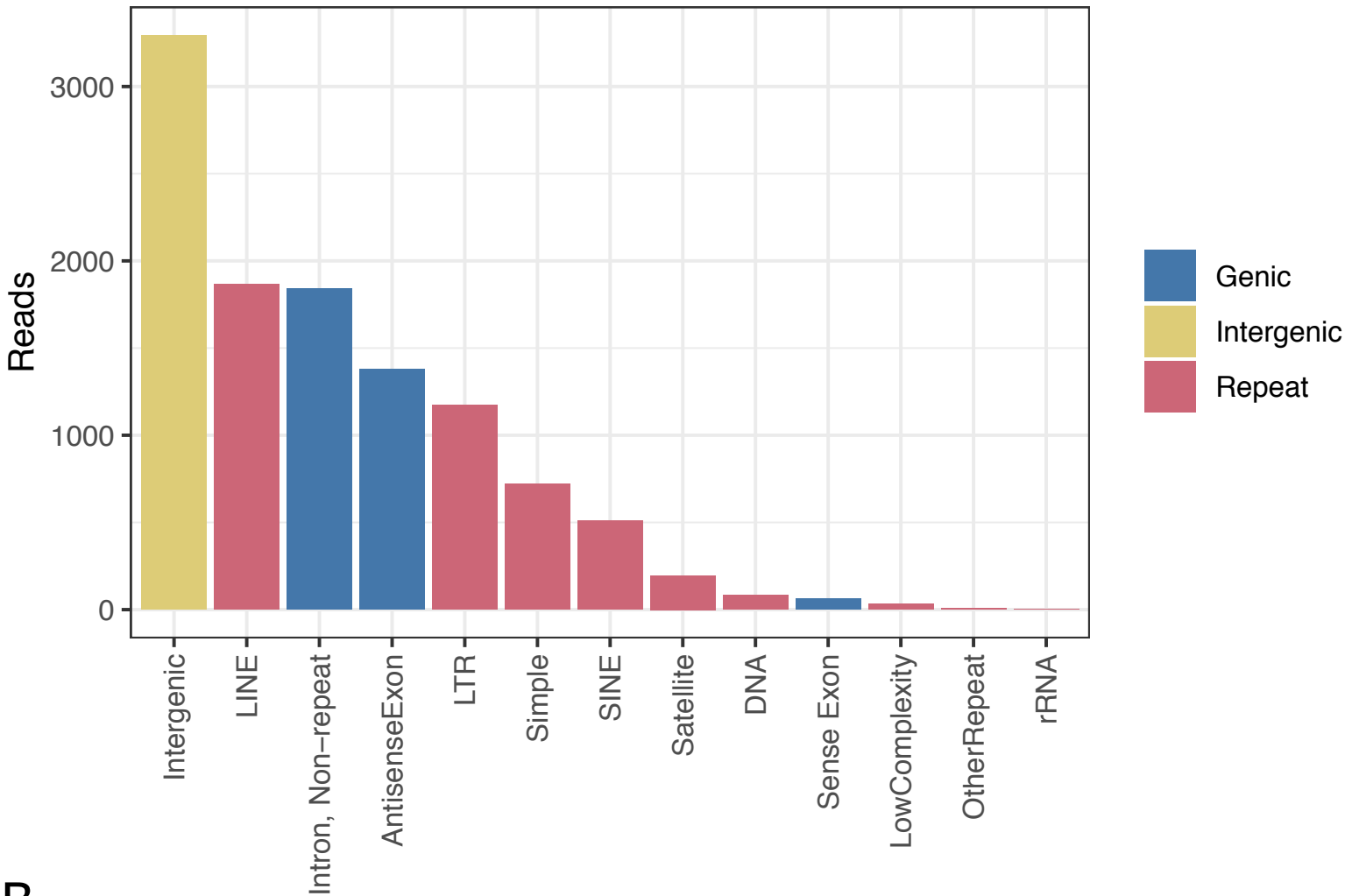

B

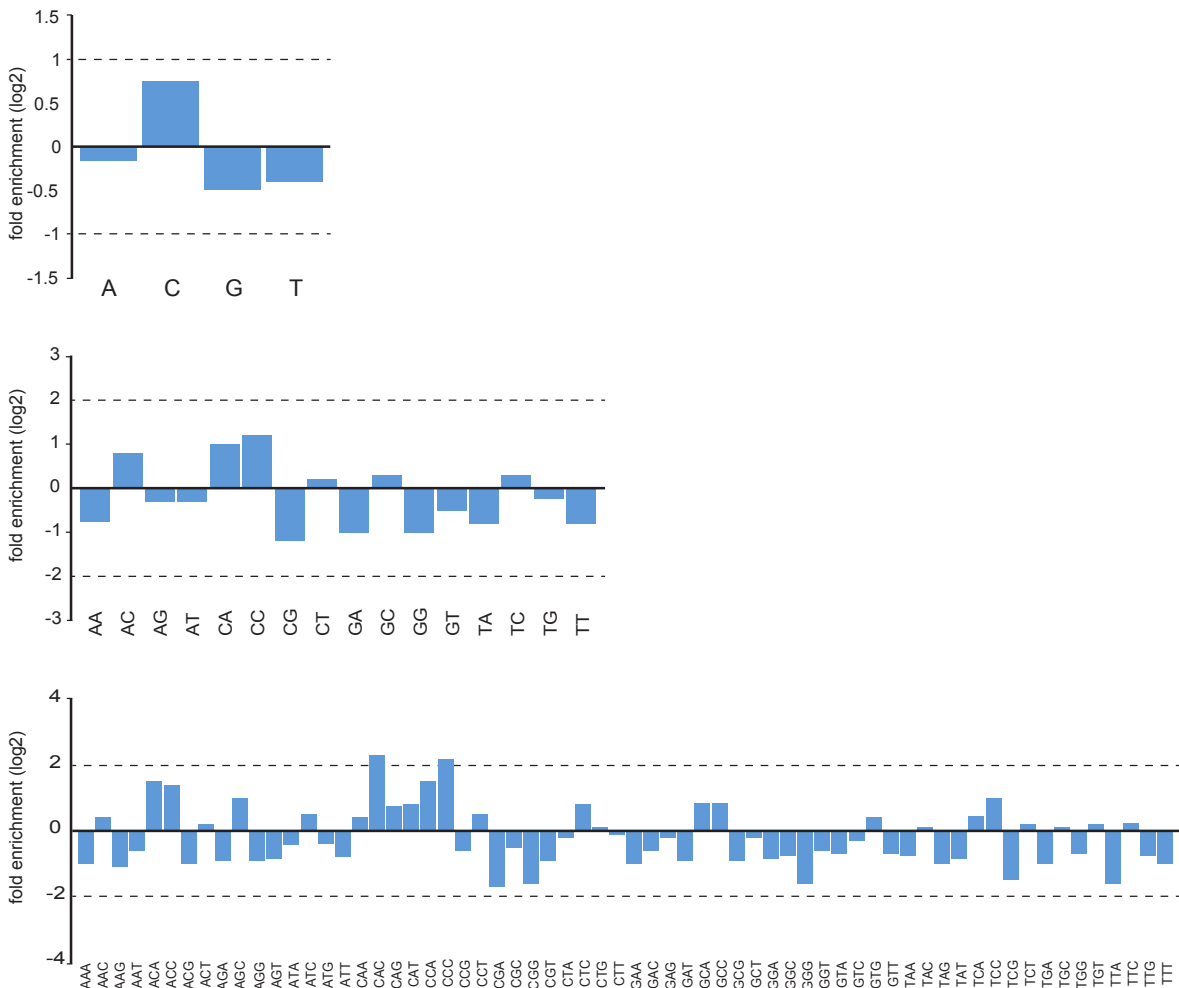

Supplement: Supplemental Material [file KTRN_A_1790990_SM0060.zip › Supplementary information/Supplementary_FigureS2 Revised.pdf]

Supplementary Figure S3

A

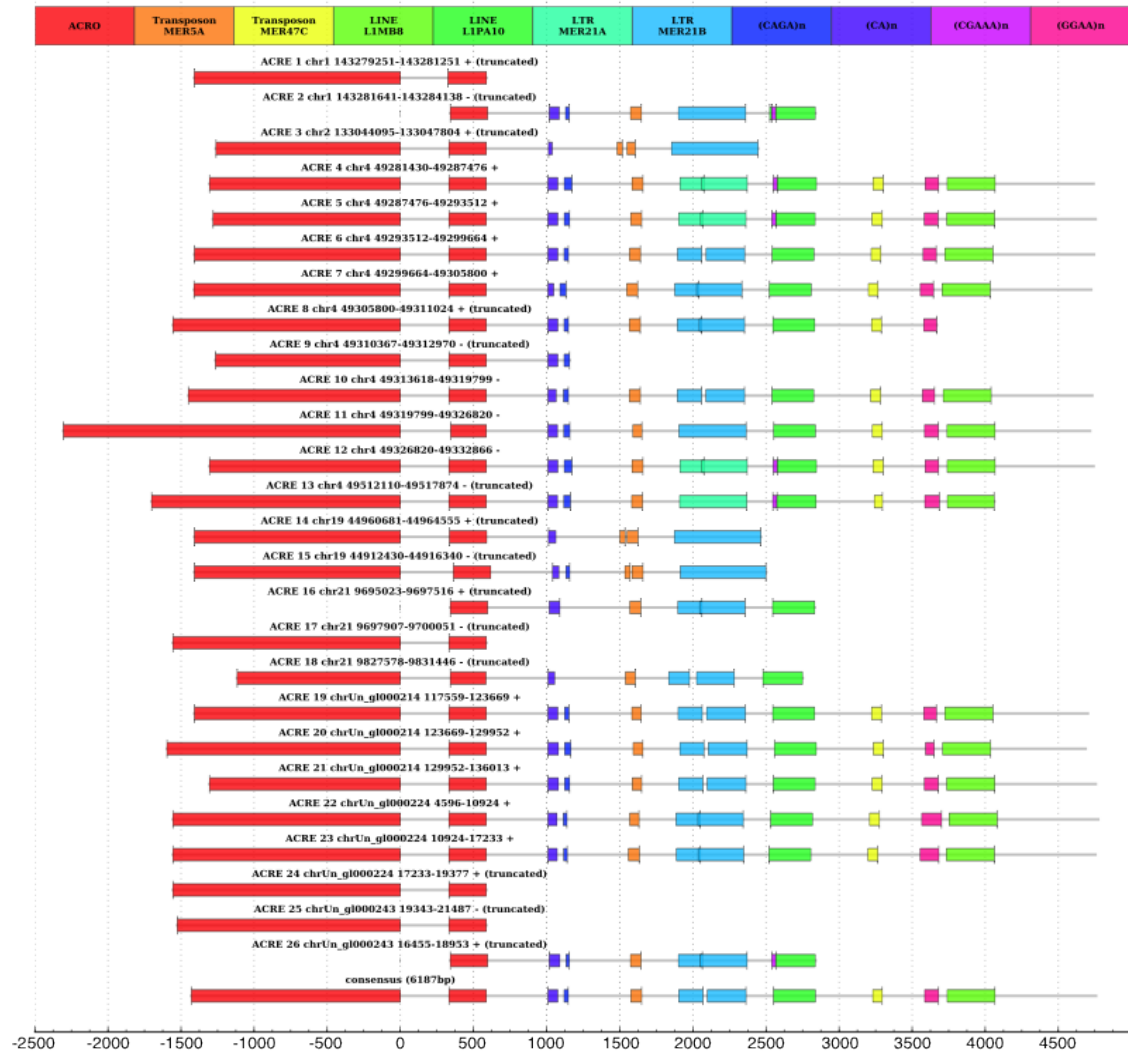

B

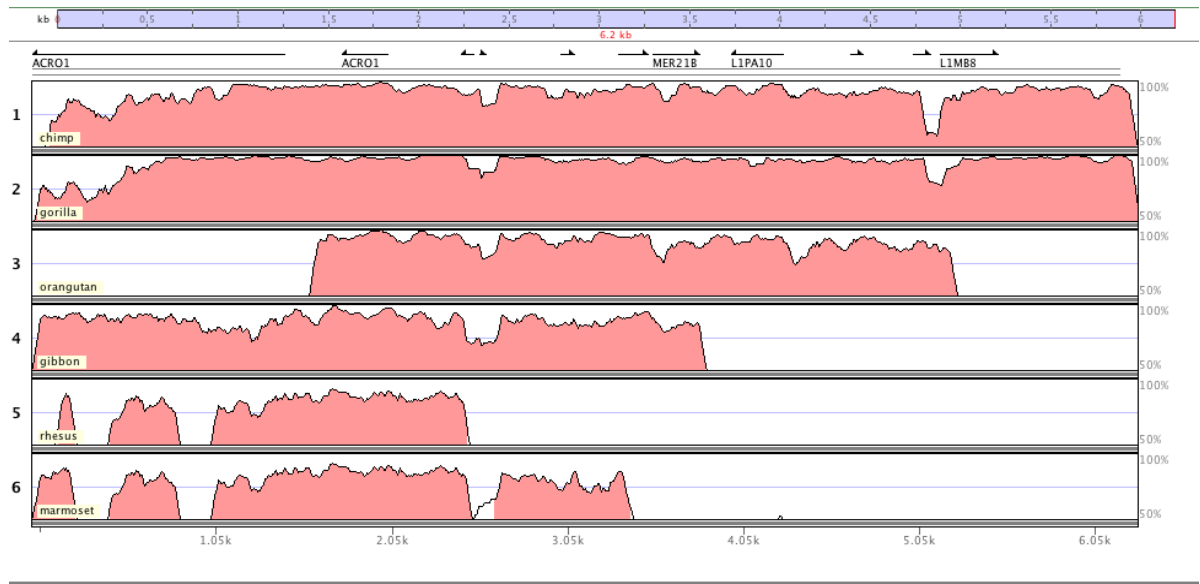

Supplement: Supplemental Material [file KTRN_A_1790990_SM0060.zip › Supplementary information/Supplementary_FigureS3.pdf]

Supplementary Figure S4

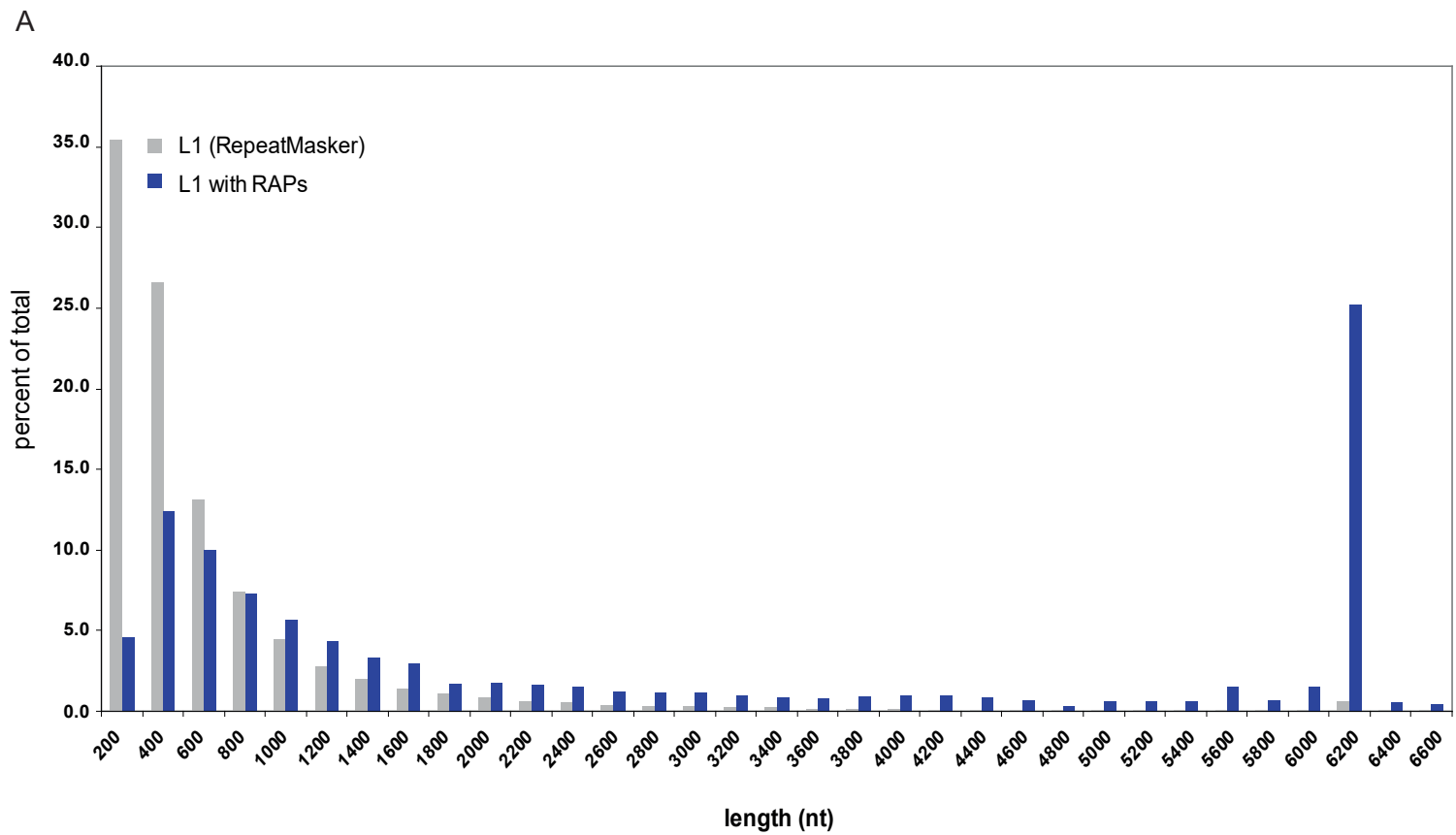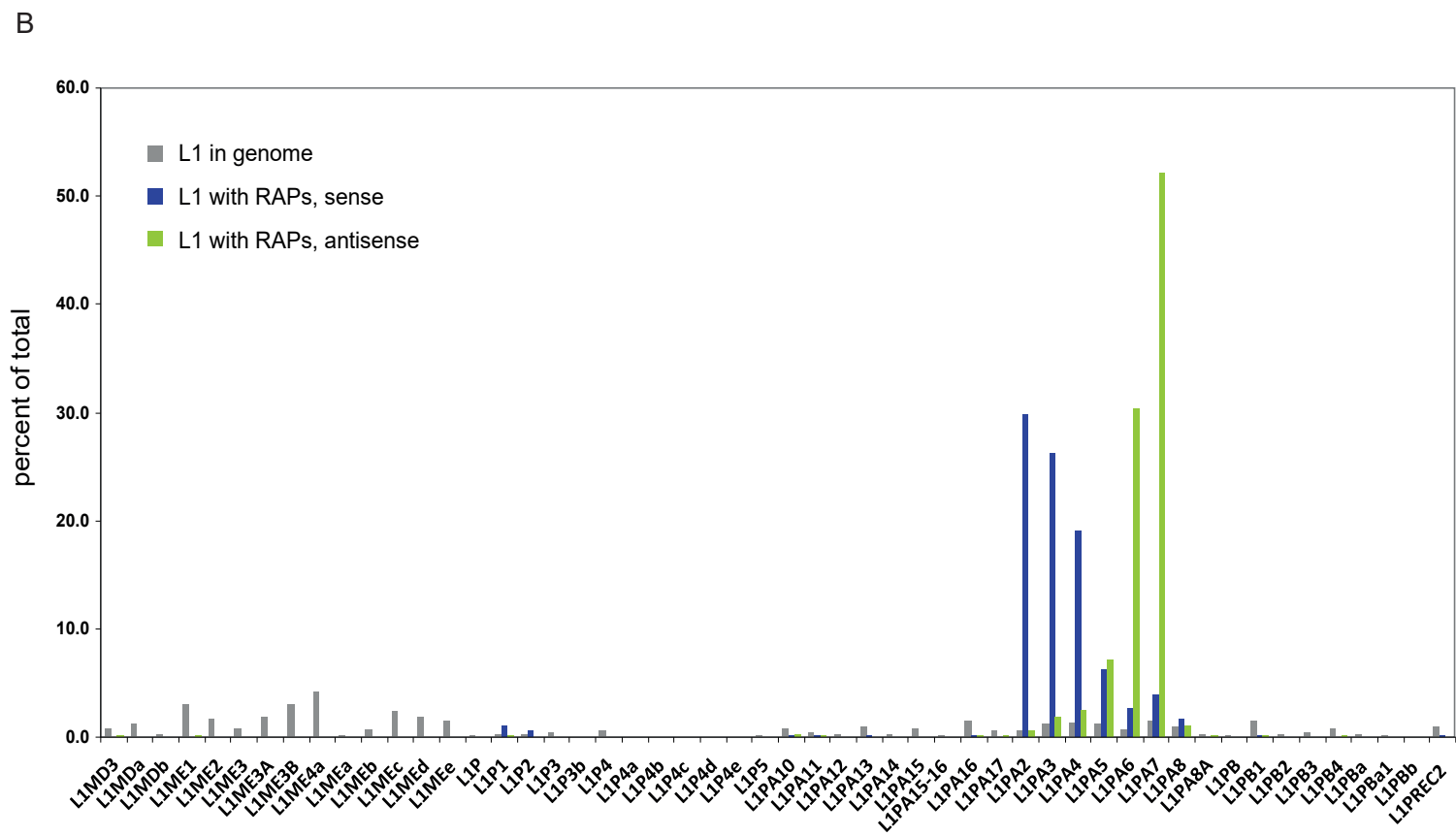

Supplement: Supplemental Material [file KTRN_A_1790990_SM0060.zip › Supplementary information/Supplementary_FigureS4.pdf]

Supplementary Figure S5

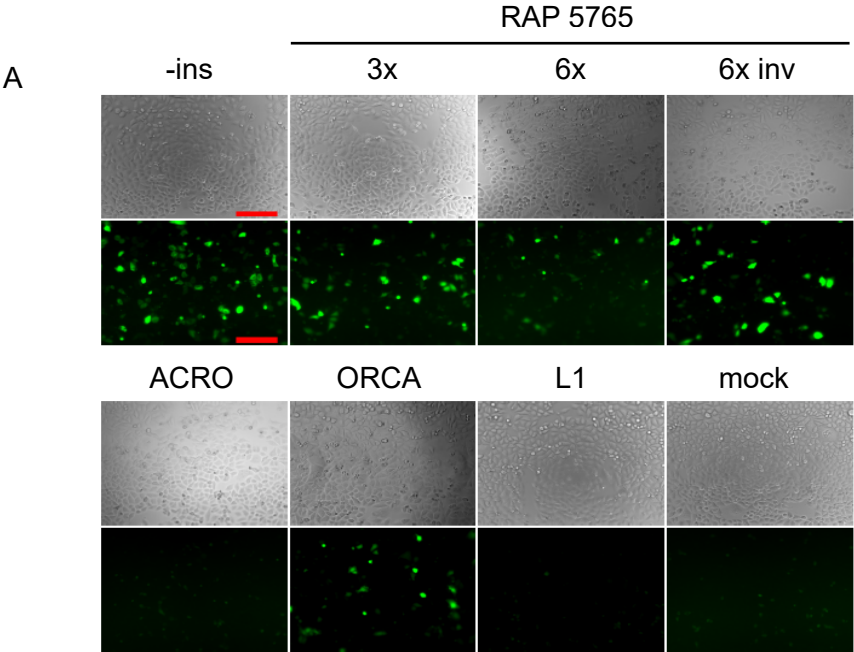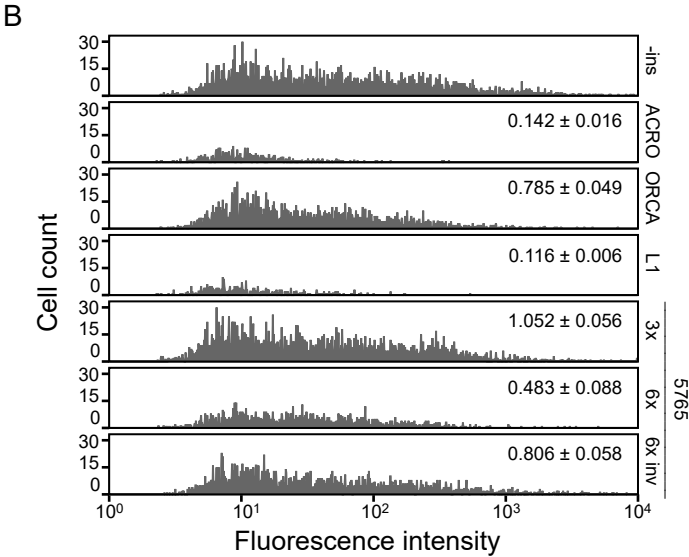

Supplement: Supplemental Material [file KTRN_A_1790990_SM0060.zip › Supplementary information/Supplementary_FigureS5.pdf]

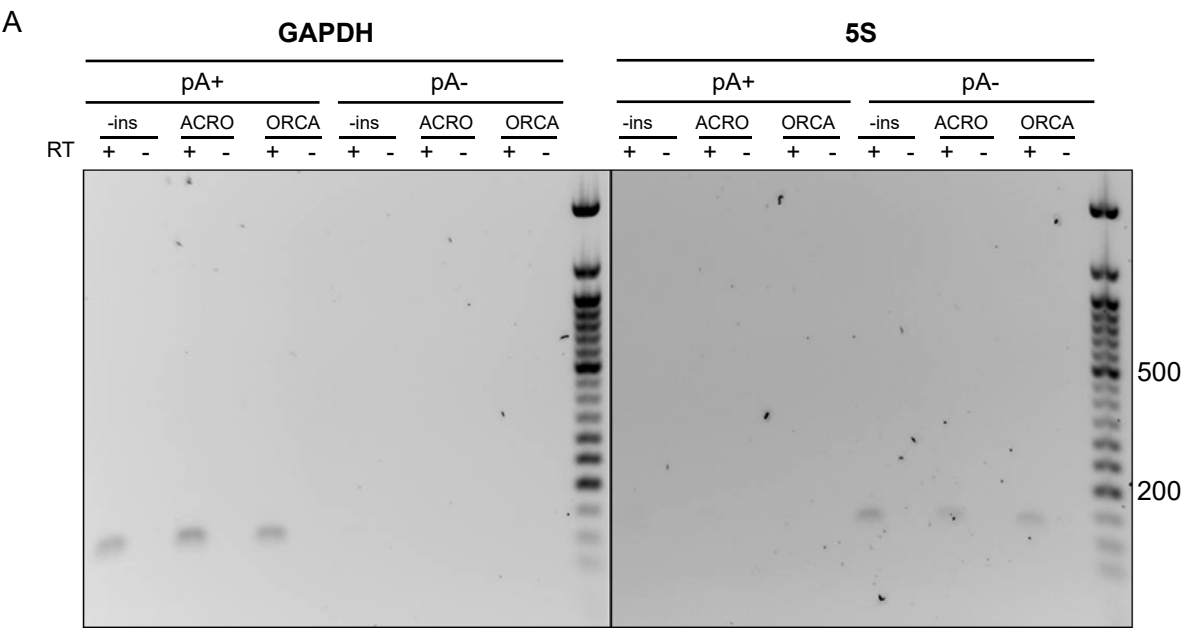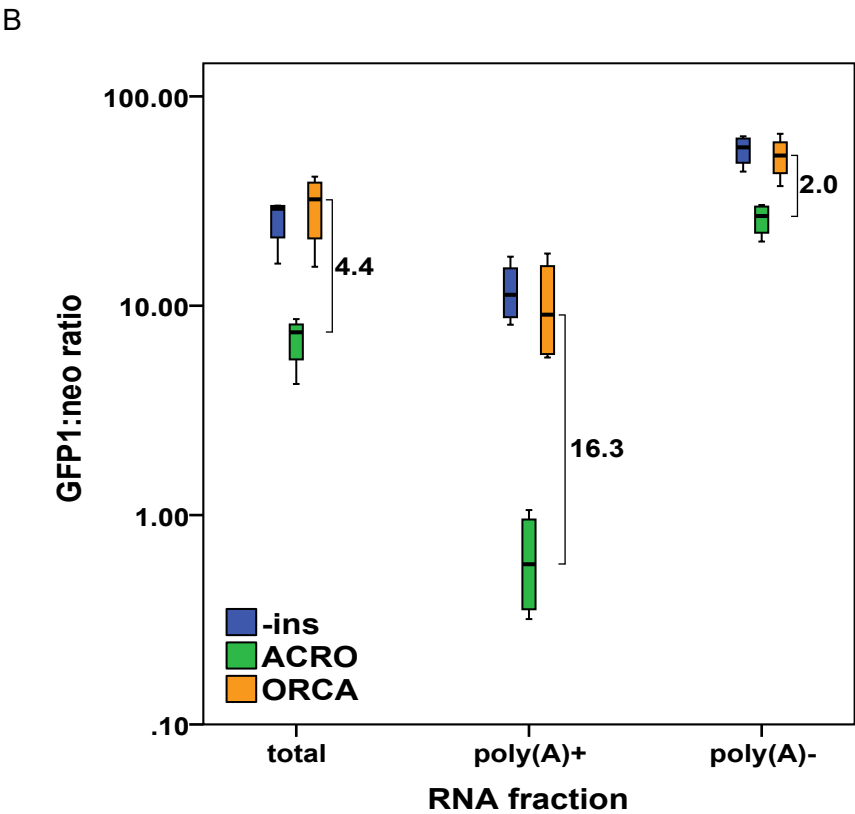

Supplement: Supplemental Material [file KTRN_A_1790990_SM0060.zip › Supplementary information/Supplementary_FigureS6.pdf]

Supplementary Figure S7

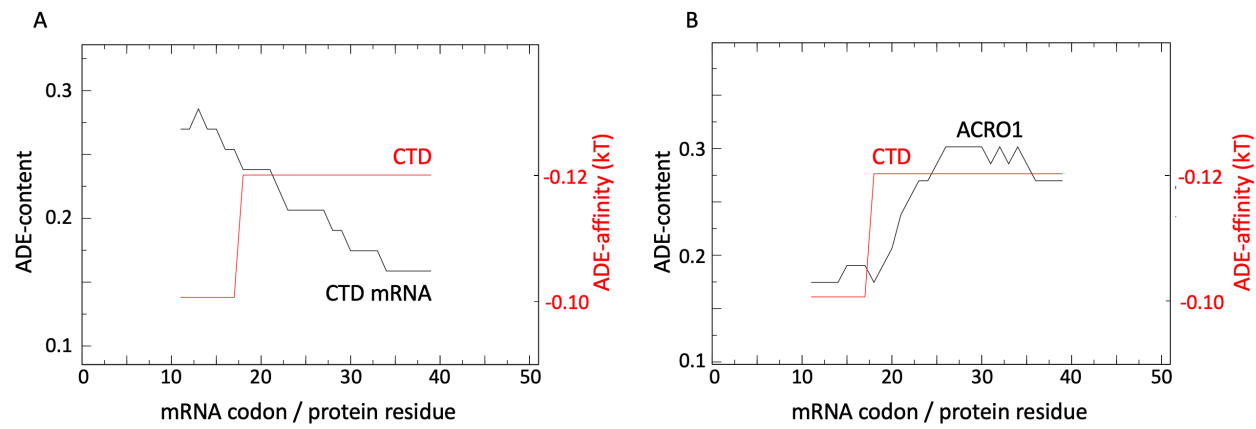

Supplement: Supplemental Material [file KTRN_A_1790990_SM0060.zip › Supplementary information/Supplementary_FigureS7.pdf]
